# Ethyl 1′-methyl-2-oxo-4′-[(3a*R*,5*R*,5a*S*,8a*S*,8b*R*)-2,2,7,7-tetra­methyl­tetra­hydro-3a*H*-bis­[1,3]dioxolo[4,5-*b*:4′,5′-*d*]pyran-5-yl]-2*H*-spiro­[ace­naphthyl­ene-1,2′-pyrrolidine]-3′-carboxyl­ate

**DOI:** 10.1107/S1600536813024586

**Published:** 2013-09-07

**Authors:** G. Jagadeesan, K. Sethusankar, R. Prasanna, R. Raghunathan

**Affiliations:** a Department of Physics, Meenakshi College of Engineering, West K.K. Nagar, Chennai 600 078, India; b Department of Physics, RKM Vivekananda College (Autonomous), Chennai 600 004, India; c Department of Organic Chemistry, University of Madras, Maraimalai Campus, Chennai 600 025, India

## Abstract

In the title mol­ecule, C_30_H_35_NO_8_, the ace­naphthyl­enone moiety, two atoms of a methyl pyrrolidine ring (N and C atoms) and four atoms of an ethyl acetate moiety (two C and two O atoms) are disordered over two sets of sites in ratio 0.532 (7):0.468 (7). The three C atoms of a di­meth­oxy­ethane ring and dioxolane ring attached with two methyl groups are disordered over two sets of sites in 0.66 (2):0.34 (2) and 0.62 (2):0.38 (2) ratios, respectively. The major and minor components of the ace­naphthyl­ene ring are essentially planar (r.m.s. deviations = 0.0254 and 0.0436 Å, respectively). The major and minor components of the pyrrolidine ring adopt C-envelope conformations with C atoms displaced by 0.492 (11) and 0.595 (7) Å from the remaining ring atoms. One of the dioxolane rings is disordered with its major component in an envelope conformation [C displaced by 0.511 (11) Å] and the minor fraction is more or less planar with an r.m.s. deviation of 0.070 Å. The other dioxolane ring is in an envelope conformation, with a C atom displaced by 0.438 (3) Å from the remainder of the ring atoms. The crystal packing features C—H⋯O inter­actions, which generate *C*(9) chains.

## Related literature

For biological properties of spiro­heterocycles, see: Kilonda *et al.* (1995 ▶); Ferguson *et al.* (2005 ▶). For a related crystal structure, see: Jagadeesan *et al.* (2012 ▶).
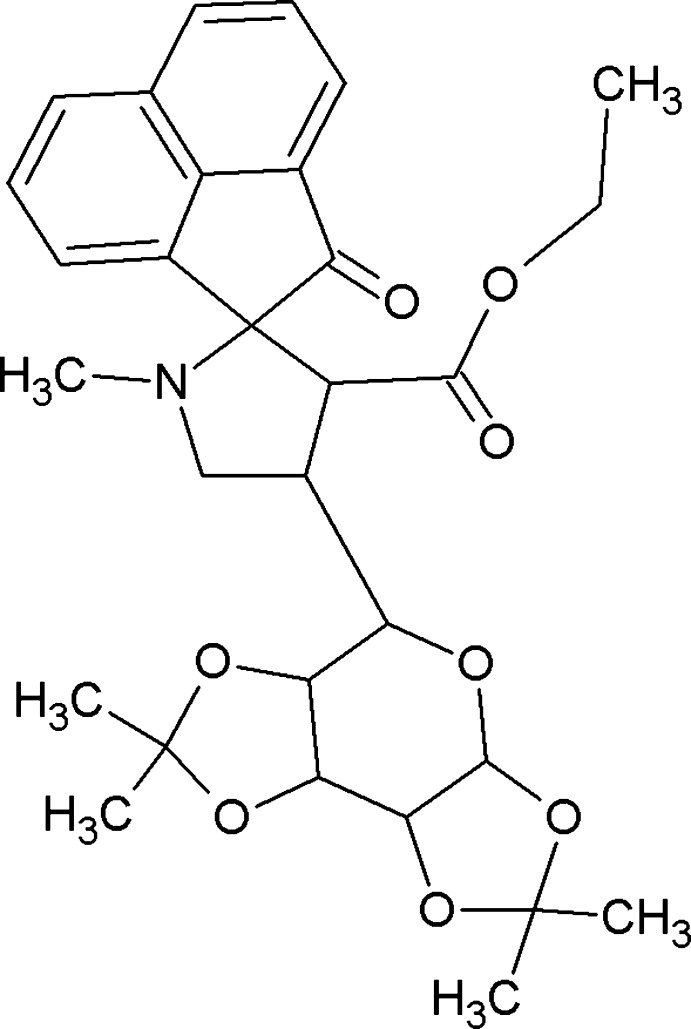


## Experimental

### 

#### Crystal data

C_30_H_35_NO_8_
*M* *_r_* = 537.59
Orthorhombic, 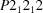

*a* = 17.3582 (15) Å
*b* = 18.5489 (13) Å
*c* = 8.9213 (8) Å
*V* = 2872.4 (4) Å^3^
*Z* = 4
Mo *K*α radiation
μ = 0.09 mm^−1^
*T* = 293 K
0.30 × 0.25 × 0.25 mm

#### Data collection

Bruker Kappa APEXII CCD diffractometer
Absorption correction: multi-scan (*SADABS*; Bruker, 2008 ▶) *T*_min_ = 0.974, *T*_max_ = 0.978
14399 measured reflections
5184 independent reflections
3771 reflections with *I* > 2σ(*I*)
*R*_int_ = 0.030

#### Refinement

*R*[*F* ^2^ > 2σ(*F* ^2^)] = 0.039
*wR*(*F* ^2^) = 0.099
*S* = 1.03
5184 reflections
572 parameters
332 restraints
H-atom parameters constrained
Δρ_max_ = 0.13 e Å^−3^
Δρ_min_ = −0.11 e Å^−3^

### 

Data collection: *APEX2* (Bruker, 2008 ▶); cell refinement: *SAINT* (Bruker, 2008 ▶); data reduction: *SAINT*; program(s) used to solve structure: *SHELXS97* (Sheldrick, 2008 ▶); program(s) used to refine structure: *SHELXL97* (Sheldrick, 2008 ▶); molecular graphics: *ORTEP-3 for Windows* (Farrugia, 2012 ▶); software used to prepare material for publication: *SHELXL97* and *PLATON* (Spek, 2009 ▶).

## Supplementary Material

Crystal structure: contains datablock(s) global, I. DOI: 10.1107/S1600536813024586/pv2644sup1.cif

Structure factors: contains datablock(s) I. DOI: 10.1107/S1600536813024586/pv2644Isup2.hkl

Additional supplementary materials:  crystallographic information; 3D view; checkCIF report

## Figures and Tables

**Table 1:** Hydrogen-bond geometry (Å, °)

*D*—H⋯*A*	*D*—H	H⋯*A*	*D*⋯*A*	*D*—H⋯*A*
C23—H23⋯O1^i^	0.98	2.45	3.324 (7)	148

Symmetry code: (i) 
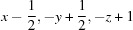
.

**Fig. 1.**
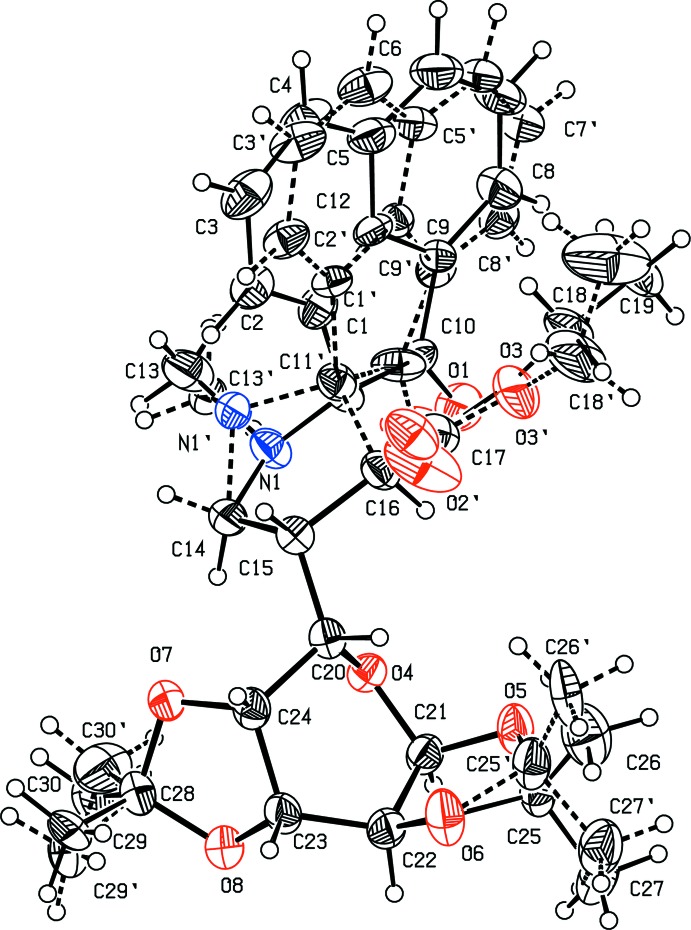
The molecular structure of the title compound with the atom numbering scheme, displacement ellipsoids are drawn at 30% probability level. H–atoms are present as small spheres of arbitary radius. The minor fractions of the disordered carbon atoms have been represented by broken bonds.

**Fig. 2.**
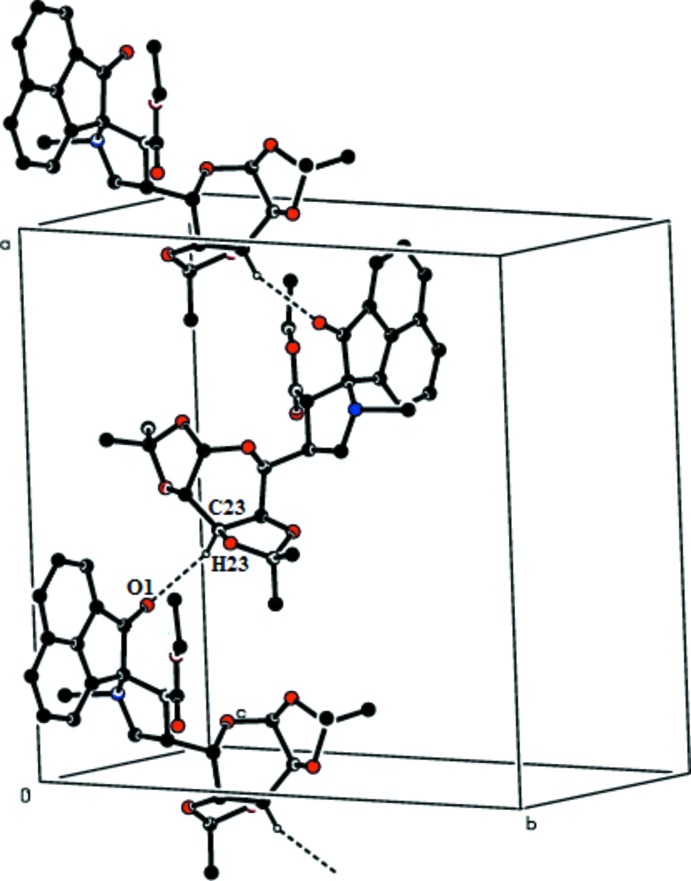
The crystal packing of the title compound viewed down c axis. Dashed lines indicates C23—H23···O1 interaction generates a C(9) chain, H–atoms not involved in hydrogen bonds have been excluded for clarity.

## supplementary crystallographic information

### 1. Comment

The design and novel synthesis of glycospiroheterocycles are interesting because of the synthetic challenges they present and their biological profile against viruses, bacteria, and cancer cells (Ferguson *et al.*, 2005). Pyrrolidines and pyrroles are common structural motifs in drugs and drug candidates owing to their ability to act as selective glycosidase inhibitors, which are used in the treatment of diabetes, cancer, malaria and viral infections, including AIDS (Kilonda *et al.*, 1995).

In the title molecule, the acenaphthylenone moiety (O1, C1–C12), two atoms of a methyl pyrrolidine ring (N1 & C11) and four atoms of an ethyl acetate moiety (O2, O3, C18 & C19) were disordered over two sites in ratio 0.531 (7):0.469 (7). The three C atoms of a dimethoxyethane ring (C25, C26 & C27) and two methyl C atoms (C29 & C30) attached to a dioxolane ring were disordered over two sites in ratios 0.659 (2):0.341 (2) and 0.615 (7): 0.385 (7), respectively. The bond distances and angles in the title compound (Fig. 1) agree very well with the corresponding bond distances and angles reported in a closely related compound (Jagadeesan *et al.*, 2012).

The major and minor components of the acenaphthylene ring (O1/C1–C12 and O1'/C1'–C12') are essentially planar (rmsd 0.0254 and 0.0436 Å, respectively). The major and minor components of pyrrolidine (C11/C14–C16/N1 & C11'/C14–C16/N1') adopt C11- and C14-envelope conformations with C atoms displaced by 0.492 (11) and 0.595 (7) °, respectively, from the remaining ring atoms. One of the dioxolane rings is disordered with major component (O5/O6/C21/C22/C25) in a C25-envelope conformation (C25 displaced by 0.511 (11) Å from the remining ring atoms) and the minor fraction (O5/O6/C21/C22/C25') is more or less planar with rmsd 0.070 Å. The other dioxolane ring (O7/O8/C23/C24/C28) is in a C28-envepole conmformation with C28 displaced by 0.438 (3) Å from the rest of the ring atoms.

The dihedral angle between the mean planes of the major acenaphthylenone moiety (C1–C12/O1) and major pyrrolidine ring (C11/C14/C15/C16/N1) is 82.91 (2)°, which shows they are almost orthogonal to each other. The mean plane of the pyran ring (C20–C24/O4) forms dihedral angles of 71.40 (2)° and 73.35 (1)° with the mean planes of the major dioxolane ring (C21/C22/C25/O5/O6) and undisordered dioxolane ring (C23/C24/C28/O7/O8), respectively.

The crystal packing is stabilized by C23—H23···O1 intermolecular interaction (Table 1 & Fig. 2) that generates a C(9) chain running along the *c*-axis.

### 2. Experimental

A mixture of (*E*)-ethyl 3-((3aR,5*R*,5aS,8aS,8bR)-2,2,7,7 -tetramethyltetrahydro-3aH-bis[1,3]dioxolo[4,5 - b:4',5'-d] pyran-5-yl)acrylate (0.915 mmol, 300 mg), acenaphthenequinone (0.915 mmol, 166 mg) and sarcosine (1.0 mmol, 97 mg) was refluxed in toluene for about 7 h under Dean stark reaction condition to give the title compound in good yield. After the completion of reaction as indicated by TLC, solvent was evaporated under reduced pressure. The crude product was purified by column chromatography using hexane: EtOAc (7:3) as eluent.

### 3. Refinement

In the acenaphthylenone moiety (C1–C12/O1), N1 and C13 atoms of the methyl pyrrolidine ring and C18/C19/O2/O3 atoms of the ethyl acetate moiety were disordered over two positions with the site occupancy factors 0.531 (7):0.469 (7). The dimethoxyethane ring C25/C26/C27 atoms and dioxolane ring attached with two methyl groups C29/C30 atoms were disordered over two positions with the site occupancy factors 0.659 (2):0.341 (2) and 0.615 (7): 0.385 (7). The bond distances of the major and minor components were restrained to a value of d(C–C) =1.39 (1) Å for aromatic ring, d(C–C) = 1.50 (1) Å for aliphatic ring and d(C–O) = 1.30 (1) Å, respectively. The bond distances of the disordered components were restrained using standard similarity restraint SADI [*SHELXL97*, Sheldrick, 2008] with s.u. of 0.01 Å. Hydrogen atoms were placed in calculated positions with C–H = 0.93 to 0.98 Å refined in the riding model with fixed isotropic displacement parameters: *U*_iso_(H) = 1.5 *U*_eq_(C) for methyl group and *U*_iso_(H) = 1.2 *U*_eq_(C) for other groups. In the absence of significant anomalous dispersion effects, an absolute structure was not deternined and 1505 Friedel pairs were merged.

### Crystal data

**Table:**

C_30_H_35_NO_8_	*F*(000) = 1144
*M**_r_* = 537.59	*D*_x_ = 1.243 Mg m^−^^3^
Orthorhombic, *P*2_1_2_1_2	Mo *K*α radiation, λ = 0.71073 Å
Hall symbol: P 2 2ab	Cell parameters from 5184 reflections
*a* = 17.3582 (15) Å	θ = 2.2–25.2°
*b* = 18.5489 (13) Å	µ = 0.09 mm^−^^1^
*c* = 8.9213 (8) Å	*T* = 293 K
*V* = 2872.4 (4) Å^3^	Block, colourless
*Z* = 4	0.30 × 0.25 × 0.25 mm

### Data collection

**Table:**

Bruker Kappa APEXII CCD diffractometer	5184 independent reflections
Radiation source: fine-focus sealed tube	3771 reflections with *I* > 2σ(*I*)
Graphite monochromator	*R*_int_ = 0.030
/w scans	θ_max_ = 25.2°, θ_min_ = 2.2°
Absorption correction: multi-scan (*SADABS*; Bruker, 2008)	*h* = −20→20
*T*_min_ = 0.974, *T*_max_ = 0.978	*k* = −13→22
14399 measured reflections	*l* = −10→6

### Refinement

**Table:**

Refinement on *F*^2^	Secondary atom site location: difference Fourier map
Least-squares matrix: full	Hydrogen site location: inferred from neighbouring sites
*R*[*F*^2^ > 2σ(*F*^2^)] = 0.039	H-atom parameters constrained
*wR*(*F*^2^) = 0.099	*w* = 1/[σ^2^(*F*_o_^2^) + (0.0476*P*)^2^ + 0.2077*P*] where *P* = (*F*_o_^2^ + 2*F*_c_^2^)/3
*S* = 1.03	(Δ/σ)_max_ = 0.004
5184 reflections	Δρ_max_ = 0.13 e Å^−^^3^
572 parameters	Δρ_min_ = −0.11 e Å^−^^3^
332 restraints	Extinction correction: *SHELXL97* (Sheldrick, 2008), Fc^*^=kFc[1+0.001xFc^2^λ^3^/sin(2θ)]^-1/4^
Primary atom site location: structure-invariant direct methods	Extinction coefficient: 0.0099 (8)

### Special details

**Table:**

Geometry. All e.s.d.'s (except the e.s.d. in the dihedral angle between two l.s. planes) are estimated using the full covariance matrix. The cell e.s.d.'s are taken into account individually in the estimation of e.s.d.'s in distances, angles and torsion angles; correlations between e.s.d.'s in cell parameters are only used when they are defined by crystal symmetry. An approximate (isotropic) treatment of cell e.s.d.'s is used for estimating e.s.d.'s involving l.s. planes.
Refinement. Refinement of *F*^2^ against ALL reflections. The weighted *R*-factor *wR* and goodness of fit *S* are based on *F*^2^, conventional *R*-factors *R* are based on *F*, with *F* set to zero for negative *F*^2^. The threshold expression of *F*^2^ > σ(*F*^2^) is used only for calculating *R*-factors(gt) *etc*. and is not relevant to the choice of reflections for refinement. *R*-factors based on *F*^2^ are statistically about twice as large as those based on *F*, and *R*- factors based on ALL data will be even larger.

### Fractional atomic coordinates and isotropic or equivalent isotropic displacement parameters (Å^2^)

**Table:**

	*x*	*y*	*z*	*U*_iso_*/*U*_eq_	Occ. (<1)
C16	0.15148 (12)	0.19659 (12)	0.2054 (3)	0.0535 (6)	
H16	0.1771	0.2119	0.2979	0.064*	
C15	0.06795 (12)	0.18045 (12)	0.2491 (3)	0.0491 (6)	
H15	0.0350	0.1872	0.1610	0.059*	
C14	0.07115 (14)	0.10032 (12)	0.2866 (3)	0.0587 (7)	
H14A	0.0572	0.0926	0.3906	0.070*	0.532 (7)
H14B	0.0353	0.0738	0.2239	0.070*	0.532 (7)
H14C	0.0574	0.0926	0.3908	0.070*	0.468 (7)
H14D	0.0351	0.0739	0.2242	0.070*	0.468 (7)
C17	0.16164 (17)	0.25778 (15)	0.0965 (4)	0.0683 (8)	
C20	0.04071 (11)	0.23067 (12)	0.3727 (3)	0.0463 (6)	
H20	0.0513	0.2802	0.3405	0.056*	
C21	0.08472 (12)	0.27183 (12)	0.6098 (3)	0.0529 (6)	
H21	0.1040	0.2532	0.7055	0.064*	
C22	0.00533 (13)	0.30468 (12)	0.6337 (3)	0.0614 (7)	
H22	−0.0038	0.3122	0.7410	0.074*	
C23	−0.06041 (12)	0.26220 (12)	0.5663 (3)	0.0572 (6)	
H23	−0.1062	0.2929	0.5581	0.069*	
C24	−0.04390 (11)	0.22622 (12)	0.4148 (3)	0.0526 (6)	
H24	−0.0753	0.2488	0.3365	0.063*	
C28	−0.10639 (14)	0.14752 (14)	0.5737 (4)	0.0681 (7)	
O4	0.08668 (8)	0.21659 (8)	0.50278 (16)	0.0480 (4)	
O5	0.13093 (8)	0.33014 (8)	0.5643 (2)	0.0675 (5)	
O6	0.00947 (8)	0.37183 (9)	0.5595 (3)	0.0797 (6)	
O7	−0.06725 (9)	0.15371 (8)	0.4351 (2)	0.0636 (5)	
O8	−0.07565 (10)	0.20380 (9)	0.6640 (2)	0.0694 (5)	
O1	0.3046 (4)	0.1229 (5)	0.3207 (9)	0.0774 (19)	0.532 (7)
C11	0.1889 (7)	0.1245 (6)	0.1623 (12)	0.049 (2)	0.532 (7)
C1	0.1841 (6)	0.1164 (10)	−0.0021 (11)	0.058 (3)	0.532 (7)
C2	0.1246 (4)	0.1115 (3)	−0.1031 (7)	0.0631 (17)	0.532 (7)
H2	0.0739	0.1129	−0.0699	0.076*	0.532 (7)
C3	0.1407 (5)	0.1041 (3)	−0.2559 (8)	0.078 (2)	0.532 (7)
H3	0.0997	0.1013	−0.3229	0.093*	0.532 (7)
C4	0.2155 (6)	0.1009 (6)	−0.3116 (12)	0.078 (3)	0.532 (7)
H4	0.2241	0.0975	−0.4143	0.093*	0.532 (7)
C5	0.2777 (5)	0.1028 (4)	−0.2117 (9)	0.0700 (19)	0.532 (7)
C6	0.3561 (6)	0.1006 (4)	−0.2442 (11)	0.087 (3)	0.532 (7)
H6	0.3708	0.0972	−0.3441	0.104*	0.532 (7)
C7	0.4139 (7)	0.1034 (8)	−0.1350 (12)	0.086 (3)	0.532 (7)
H7	0.4655	0.1019	−0.1631	0.103*	0.532 (7)
C8	0.3941 (5)	0.1083 (5)	0.0151 (11)	0.077 (2)	0.532 (7)
H8	0.4324	0.1092	0.0880	0.092*	0.532 (7)
C9	0.3175 (6)	0.1119 (10)	0.0562 (14)	0.052 (3)	0.532 (7)
C12	0.2596 (7)	0.1098 (10)	−0.057 (2)	0.056 (3)	0.532 (7)
C10	0.2752 (5)	0.1185 (8)	0.1970 (10)	0.047 (2)	0.532 (7)
N1	0.1465 (3)	0.0764 (2)	0.2608 (7)	0.0540 (14)	0.532 (7)
C13	0.1440 (10)	−0.0046 (9)	0.1778 (15)	0.082 (4)	0.532 (7)
H13A	0.1957	−0.0208	0.1591	0.124*	0.532 (7)
H13B	0.1167	−0.0010	0.0845	0.124*	0.532 (7)
H13C	0.1184	−0.0384	0.2421	0.124*	0.532 (7)
O2	0.1136 (5)	0.2854 (7)	0.0177 (18)	0.079 (2)	0.532 (7)
O3	0.2367 (3)	0.2687 (6)	0.0732 (13)	0.056 (2)	0.532 (7)
C18	0.2624 (5)	0.3178 (6)	−0.0435 (13)	0.074 (2)	0.532 (7)
H18A	0.2471	0.3668	−0.0201	0.088*	0.532 (7)
H18B	0.2402	0.3045	−0.1394	0.088*	0.532 (7)
C19	0.3474 (4)	0.3117 (7)	−0.0484 (11)	0.084 (3)	0.532 (7)
H19A	0.3676	0.3455	−0.1198	0.125*	0.532 (7)
H19B	0.3615	0.2637	−0.0775	0.125*	0.532 (7)
H19C	0.3682	0.3221	0.0489	0.125*	0.532 (7)
O1'	0.2724 (5)	0.1030 (5)	0.3368 (9)	0.085 (3)	0.468 (7)
C11'	0.1821 (9)	0.1253 (7)	0.1322 (13)	0.049 (3)	0.468 (7)
C1'	0.2002 (6)	0.1177 (11)	−0.0318 (10)	0.044 (2)	0.468 (7)
C2'	0.1593 (4)	0.1205 (4)	−0.1638 (9)	0.0592 (19)	0.468 (7)
H2'	0.1063	0.1274	−0.1588	0.071*	0.468 (7)
C3'	0.1933 (6)	0.1136 (8)	−0.3043 (12)	0.067 (3)	0.468 (7)
H3'	0.1627	0.1117	−0.3898	0.080*	0.468 (7)
C4'	0.2739 (5)	0.1096 (4)	−0.3166 (8)	0.069 (2)	0.468 (7)
H4'	0.2965	0.1088	−0.4111	0.082*	0.468 (7)
C5'	0.3204 (5)	0.1068 (4)	−0.1898 (9)	0.0534 (18)	0.468 (7)
C6'	0.4005 (6)	0.1004 (8)	−0.1857 (11)	0.058 (2)	0.468 (7)
H6'	0.4284	0.0996	−0.2746	0.069*	0.468 (7)
C7'	0.4388 (4)	0.0952 (4)	−0.0480 (9)	0.0644 (19)	0.468 (7)
H7'	0.4922	0.0911	−0.0474	0.077*	0.468 (7)
C8'	0.3991 (5)	0.0959 (4)	0.0894 (8)	0.0536 (18)	0.468 (7)
H8'	0.4253	0.0905	0.1797	0.064*	0.468 (7)
C9'	0.3192 (7)	0.1050 (12)	0.0864 (16)	0.051 (3)	0.468 (7)
C12'	0.2810 (6)	0.1100 (11)	−0.054 (2)	0.042 (2)	0.468 (7)
C10'	0.2599 (8)	0.1107 (11)	0.1996 (11)	0.064 (3)	0.468 (7)
N1'	0.1207 (3)	0.0695 (2)	0.1654 (8)	0.0585 (17)	0.468 (7)
C13'	0.1557 (12)	0.0010 (11)	0.2335 (15)	0.058 (3)	0.468 (7)
H13D	0.1884	−0.0219	0.1609	0.087*	0.468 (7)
H13E	0.1152	−0.0315	0.2617	0.087*	0.468 (7)
H13F	0.1854	0.0135	0.3204	0.087*	0.468 (7)
O2'	0.1099 (8)	0.2973 (9)	0.059 (2)	0.129 (8)	0.468 (7)
O3'	0.2356 (4)	0.2741 (9)	0.085 (2)	0.105 (5)	0.468 (7)
C18'	0.2517 (10)	0.3333 (8)	−0.016 (2)	0.129 (5)	0.468 (7)
H18C	0.2634	0.3768	0.0393	0.155*	0.468 (7)
H18D	0.2079	0.3425	−0.0811	0.155*	0.468 (7)
C19'	0.3189 (10)	0.3102 (9)	−0.1052 (19)	0.170 (7)	0.468 (7)
H19D	0.3291	0.3451	−0.1821	0.256*	0.468 (7)
H19E	0.3084	0.2642	−0.1504	0.256*	0.468 (7)
H19F	0.3631	0.3061	−0.0408	0.256*	0.468 (7)
C25	0.0875 (2)	0.3946 (3)	0.5826 (13)	0.061 (2)	0.66 (2)
C26	0.1071 (7)	0.4412 (6)	0.4537 (17)	0.101 (3)	0.66 (2)
H26A	0.1591	0.4583	0.4640	0.152*	0.66 (2)
H26B	0.0725	0.4816	0.4507	0.152*	0.66 (2)
H26C	0.1025	0.4141	0.3625	0.152*	0.66 (2)
C27	0.1041 (6)	0.4301 (5)	0.7319 (14)	0.085 (3)	0.66 (2)
H27A	0.1573	0.4440	0.7361	0.128*	0.66 (2)
H27B	0.0931	0.3969	0.8115	0.128*	0.66 (2)
H27C	0.0723	0.4722	0.7428	0.128*	0.66 (2)
C25'	0.0876 (4)	0.3935 (6)	0.528 (2)	0.070 (4)	0.34 (2)
C26'	0.1038 (12)	0.4293 (11)	0.382 (3)	0.087 (5)	0.34 (2)
H26D	0.1576	0.4408	0.3754	0.131*	0.34 (2)
H26E	0.0741	0.4728	0.3743	0.131*	0.34 (2)
H26F	0.0901	0.3975	0.3011	0.131*	0.34 (2)
C27'	0.0992 (11)	0.4457 (10)	0.656 (3)	0.090 (4)	0.34 (2)
H27D	0.1500	0.4660	0.6503	0.136*	0.34 (2)
H27E	0.0933	0.4206	0.7495	0.136*	0.34 (2)
H27F	0.0617	0.4836	0.6497	0.136*	0.34 (2)
C29	−0.1910 (4)	0.1618 (14)	0.539 (3)	0.087 (4)	0.62 (7)
H29A	−0.2208	0.1581	0.6289	0.130*	0.62 (7)
H29B	−0.2090	0.1269	0.4672	0.130*	0.62 (7)
H29C	−0.1965	0.2093	0.4973	0.130*	0.62 (7)
C30	−0.0907 (13)	0.0786 (8)	0.658 (3)	0.099 (5)	0.62 (7)
H30A	−0.1189	0.0788	0.7506	0.148*	0.62 (7)
H30B	−0.0365	0.0749	0.6789	0.148*	0.62 (7)
H30C	−0.1066	0.0382	0.5984	0.148*	0.62 (7)
C29'	−0.1936 (5)	0.151 (3)	0.576 (6)	0.114 (9)	0.38 (7)
H29D	−0.2117	0.1456	0.6769	0.171*	0.38 (7)
H29E	−0.2143	0.1135	0.5145	0.171*	0.38 (7)
H29F	−0.2101	0.1972	0.5377	0.171*	0.38 (7)
C30'	−0.076 (3)	0.0750 (12)	0.623 (5)	0.102 (7)	0.38 (7)
H30D	−0.0961	0.0639	0.7209	0.153*	0.38 (7)
H30E	−0.0210	0.0763	0.6266	0.153*	0.38 (7)
H30F	−0.0926	0.0387	0.5533	0.153*	0.38 (7)

### Atomic displacement parameters (Å^2^)

**Table:**

	*U*^11^	*U*^22^	*U*^33^	*U*^12^	*U*^13^	*U*^23^
C16	0.0525 (13)	0.0476 (14)	0.0605 (16)	−0.0024 (10)	0.0128 (12)	0.0054 (11)
C15	0.0474 (12)	0.0501 (14)	0.0497 (14)	−0.0003 (10)	0.0018 (12)	0.0016 (11)
C14	0.0614 (15)	0.0507 (15)	0.0639 (16)	−0.0090 (11)	0.0201 (13)	−0.0079 (12)
C17	0.0784 (19)	0.0495 (17)	0.077 (2)	0.0011 (14)	0.0238 (18)	0.0040 (14)
C20	0.0404 (11)	0.0421 (12)	0.0565 (15)	0.0036 (9)	−0.0029 (11)	0.0053 (11)
C21	0.0498 (12)	0.0470 (14)	0.0621 (16)	−0.0004 (10)	−0.0026 (12)	−0.0089 (11)
C22	0.0536 (13)	0.0470 (15)	0.0836 (19)	0.0000 (10)	0.0104 (14)	−0.0166 (13)
C23	0.0389 (11)	0.0485 (14)	0.0841 (18)	0.0018 (9)	0.0092 (13)	−0.0101 (14)
C24	0.0403 (11)	0.0522 (14)	0.0652 (16)	0.0022 (10)	−0.0047 (12)	−0.0021 (12)
C28	0.0573 (14)	0.0652 (17)	0.082 (2)	−0.0200 (12)	0.0146 (15)	−0.0097 (15)
O4	0.0451 (8)	0.0437 (9)	0.0551 (9)	0.0092 (6)	−0.0045 (7)	−0.0042 (7)
O5	0.0417 (8)	0.0460 (9)	0.1148 (15)	−0.0002 (7)	−0.0007 (10)	−0.0064 (10)
O6	0.0441 (9)	0.0443 (10)	0.1506 (18)	0.0040 (7)	0.0031 (12)	−0.0044 (11)
O7	0.0514 (9)	0.0599 (11)	0.0795 (12)	−0.0121 (7)	0.0149 (10)	−0.0162 (9)
O8	0.0685 (11)	0.0665 (12)	0.0732 (12)	−0.0185 (9)	0.0115 (10)	−0.0105 (10)
O1	0.067 (4)	0.104 (4)	0.061 (4)	−0.002 (3)	−0.015 (3)	−0.007 (3)
C11	0.048 (5)	0.050 (4)	0.050 (4)	0.002 (3)	0.011 (3)	0.011 (3)
C1	0.070 (5)	0.042 (3)	0.061 (5)	0.000 (4)	−0.009 (4)	0.000 (4)
C2	0.080 (4)	0.054 (3)	0.055 (4)	−0.002 (3)	−0.004 (3)	−0.002 (3)
C3	0.116 (5)	0.067 (3)	0.051 (4)	−0.002 (4)	−0.020 (4)	−0.002 (3)
C4	0.129 (7)	0.064 (5)	0.039 (4)	−0.002 (6)	0.001 (5)	−0.010 (3)
C5	0.111 (5)	0.044 (3)	0.055 (4)	−0.004 (4)	0.027 (4)	−0.005 (3)
C6	0.119 (7)	0.062 (4)	0.080 (5)	−0.002 (4)	0.047 (5)	−0.014 (4)
C7	0.085 (6)	0.084 (5)	0.089 (7)	−0.001 (5)	0.046 (6)	−0.007 (6)
C8	0.065 (4)	0.079 (4)	0.087 (6)	−0.005 (3)	0.023 (5)	−0.012 (5)
C9	0.047 (4)	0.051 (5)	0.057 (6)	−0.004 (3)	0.018 (4)	−0.017 (4)
C12	0.092 (6)	0.039 (3)	0.038 (4)	−0.005 (6)	0.014 (6)	−0.006 (3)
C10	0.040 (3)	0.055 (4)	0.045 (5)	0.005 (3)	−0.013 (3)	−0.013 (3)
N1	0.051 (2)	0.048 (2)	0.064 (3)	0.0023 (17)	0.014 (2)	0.010 (2)
C13	0.097 (9)	0.040 (5)	0.110 (11)	0.007 (5)	0.055 (8)	0.005 (7)
O2	0.070 (4)	0.074 (4)	0.093 (5)	0.013 (3)	0.018 (4)	0.032 (4)
O3	0.060 (4)	0.049 (4)	0.057 (4)	−0.006 (3)	0.017 (3)	0.007 (3)
C18	0.080 (4)	0.049 (4)	0.092 (5)	0.004 (3)	0.045 (4)	0.017 (4)
C19	0.082 (4)	0.094 (5)	0.075 (6)	−0.021 (4)	0.031 (4)	0.025 (4)
O1'	0.092 (6)	0.126 (7)	0.036 (3)	0.042 (5)	0.003 (4)	0.009 (3)
C11'	0.050 (5)	0.046 (4)	0.050 (6)	0.000 (3)	0.005 (4)	0.006 (4)
C1'	0.054 (5)	0.047 (4)	0.032 (4)	0.008 (4)	−0.007 (4)	−0.006 (3)
C2'	0.064 (4)	0.065 (4)	0.048 (4)	0.014 (3)	−0.015 (4)	−0.010 (4)
C3'	0.089 (6)	0.070 (5)	0.041 (5)	0.034 (4)	−0.020 (4)	−0.002 (4)
C4'	0.104 (5)	0.062 (4)	0.039 (4)	0.017 (4)	−0.001 (4)	0.004 (3)
C5'	0.065 (4)	0.048 (3)	0.048 (4)	0.010 (4)	0.013 (4)	0.002 (3)
C6'	0.058 (5)	0.063 (4)	0.052 (5)	0.003 (4)	0.017 (4)	−0.007 (4)
C7'	0.060 (4)	0.070 (4)	0.063 (5)	0.000 (3)	0.014 (4)	−0.007 (3)
C8'	0.066 (4)	0.046 (3)	0.049 (4)	−0.003 (3)	−0.003 (4)	−0.001 (3)
C9'	0.065 (5)	0.041 (4)	0.047 (5)	0.000 (4)	−0.002 (4)	0.002 (4)
C12'	0.059 (4)	0.033 (4)	0.034 (4)	0.004 (4)	0.010 (4)	0.001 (3)
C10'	0.085 (7)	0.072 (6)	0.036 (5)	0.016 (6)	0.015 (4)	0.008 (4)
N1'	0.061 (3)	0.043 (3)	0.072 (4)	−0.010 (2)	0.020 (3)	−0.013 (3)
C13'	0.072 (5)	0.042 (4)	0.060 (7)	−0.011 (3)	0.014 (5)	−0.009 (5)
O2'	0.132 (8)	0.100 (8)	0.155 (15)	0.055 (6)	0.069 (7)	0.067 (9)
O3'	0.116 (8)	0.082 (8)	0.117 (9)	−0.040 (6)	0.047 (6)	0.008 (6)
C18'	0.164 (9)	0.085 (8)	0.139 (9)	−0.023 (7)	0.088 (7)	0.021 (6)
C19'	0.273 (17)	0.091 (8)	0.147 (13)	0.052 (10)	0.113 (12)	0.041 (8)
C25	0.045 (3)	0.041 (3)	0.098 (6)	−0.003 (2)	0.003 (2)	−0.004 (3)
C26	0.083 (5)	0.080 (5)	0.141 (9)	−0.003 (4)	−0.003 (5)	0.024 (5)
C27	0.081 (4)	0.063 (4)	0.112 (7)	−0.004 (3)	−0.019 (5)	−0.027 (4)
C25'	0.044 (5)	0.049 (6)	0.115 (10)	0.004 (4)	−0.008 (5)	−0.009 (5)
C26'	0.050 (5)	0.063 (7)	0.148 (13)	−0.025 (5)	−0.018 (9)	0.034 (9)
C27'	0.061 (6)	0.085 (8)	0.125 (12)	−0.003 (5)	−0.016 (8)	−0.022 (8)
C29	0.041 (4)	0.120 (7)	0.099 (9)	−0.026 (4)	0.030 (3)	−0.033 (7)
C30	0.114 (6)	0.066 (5)	0.117 (13)	−0.023 (4)	0.036 (6)	0.000 (5)
C29'	0.073 (9)	0.147 (15)	0.122 (17)	−0.042 (8)	0.020 (8)	−0.019 (13)
C30'	0.159 (17)	0.074 (8)	0.072 (12)	−0.011 (8)	0.019 (13)	0.011 (7)

### Geometric parameters (Å, º)

**Table:**

C16—C17	1.504 (4)	O3—C18	1.454 (6)
C16—C15	1.531 (3)	C18—C19	1.480 (8)
C16—C11	1.535 (8)	C18—H18A	0.9700
C16—C11'	1.568 (9)	C18—H18B	0.9700
C16—H16	0.9800	C19—H19A	0.9600
C15—C20	1.519 (3)	C19—H19B	0.9600
C15—C14	1.524 (3)	C19—H19C	0.9600
C15—H15	0.9800	O1'—C10'	1.251 (9)
C14—N1	1.400 (4)	C11'—C10'	1.503 (9)
C14—N1'	1.496 (6)	C11'—C1'	1.503 (9)
C14—H14A	0.9700	C11'—N1'	1.515 (18)
C14—H14B	0.9700	C1'—C2'	1.376 (8)
C14—H14C	0.9700	C1'—C12'	1.422 (9)
C14—H14D	0.9700	C2'—C3'	1.391 (9)
C17—O2	1.204 (7)	C2'—H2'	0.9300
C17—O2'	1.208 (8)	C3'—C4'	1.406 (9)
C17—O3'	1.323 (7)	C3'—H3'	0.9300
C17—O3	1.335 (6)	C4'—C5'	1.391 (8)
C20—O4	1.433 (2)	C4'—H4'	0.9300
C20—C24	1.518 (3)	C5'—C6'	1.395 (9)
C20—H20	0.9800	C5'—C12'	1.40 (2)
C21—O4	1.401 (3)	C6'—C7'	1.401 (8)
C21—O5	1.406 (3)	C6'—H6'	0.9300
C21—C22	1.522 (3)	C7'—C8'	1.407 (8)
C21—H21	0.9800	C7'—H7'	0.9300
C22—O6	1.412 (3)	C8'—C9'	1.398 (9)
C22—C23	1.512 (3)	C8'—H8'	0.9300
C22—H22	0.9800	C9'—C12'	1.42 (2)
C23—O8	1.415 (3)	C9'—C10'	1.445 (9)
C23—C24	1.534 (3)	N1'—C13'	1.53 (2)
C23—H23	0.9800	C13'—H13D	0.9600
C24—O7	1.416 (3)	C13'—H13E	0.9600
C24—H24	0.9800	C13'—H13F	0.9600
C28—O7	1.415 (3)	O3'—C18'	1.447 (8)
C28—O8	1.423 (3)	C18'—C19'	1.474 (10)
C28—C30	1.509 (7)	C18'—H18C	0.9700
C28—C30'	1.508 (9)	C18'—H18D	0.9700
C28—C29'	1.516 (9)	C19'—H19D	0.9600
C28—C29	1.525 (7)	C19'—H19E	0.9600
O5—C25	1.423 (5)	C19'—H19F	0.9600
O5—C25'	1.433 (8)	C25—C26	1.479 (8)
O6—C25	1.435 (5)	C25—C27	1.513 (7)
O6—C25'	1.442 (8)	C26—H26A	0.9600
O1—C10	1.219 (8)	C26—H26B	0.9600
C11—N1	1.453 (15)	C26—H26C	0.9600
C11—C1	1.477 (8)	C27—H27A	0.9600
C11—C10	1.533 (9)	C27—H27B	0.9600
C1—C2	1.373 (8)	C27—H27C	0.9600
C1—C12	1.405 (9)	C25'—C26'	1.489 (11)
C2—C3	1.398 (7)	C25'—C27'	1.513 (10)
C2—H2	0.9300	C26'—H26D	0.9600
C3—C4	1.392 (8)	C26'—H26E	0.9600
C3—H3	0.9300	C26'—H26F	0.9600
C4—C5	1.401 (9)	C27'—H27D	0.9600
C4—H4	0.9300	C27'—H27E	0.9600
C5—C6	1.391 (8)	C27'—H27F	0.9600
C5—C12	1.42 (2)	C29—H29A	0.9600
C6—C7	1.400 (9)	C29—H29B	0.9600
C6—H6	0.9300	C29—H29C	0.9600
C7—C8	1.385 (9)	C30—H30A	0.9600
C7—H7	0.9300	C30—H30B	0.9600
C8—C9	1.381 (8)	C30—H30C	0.9600
C8—H8	0.9300	C29'—H29D	0.9600
C9—C12	1.43 (2)	C29'—H29E	0.9600
C9—C10	1.461 (8)	C29'—H29F	0.9600
N1—C13	1.675 (16)	C30'—H30D	0.9600
C13—H13A	0.9600	C30'—H30E	0.9600
C13—H13B	0.9600	C30'—H30F	0.9600
C13—H13C	0.9600		
			
C17—C16—C15	115.0 (2)	H13A—C13—H13C	109.5
C17—C16—C11	116.5 (5)	H13B—C13—H13C	109.5
C15—C16—C11	107.1 (6)	C17—O3—C18	120.4 (7)
C17—C16—C11'	109.2 (6)	O3—C18—C19	106.2 (7)
C15—C16—C11'	105.2 (7)	O3—C18—H18A	110.5
C17—C16—H16	105.8	C19—C18—H18A	110.5
C15—C16—H16	105.8	O3—C18—H18B	110.5
C11—C16—H16	105.8	C19—C18—H18B	110.5
C11'—C16—H16	116.2	H18A—C18—H18B	108.7
C20—C15—C14	116.78 (19)	C18—C19—H19A	109.5
C20—C15—C16	111.10 (19)	C18—C19—H19B	109.5
C14—C15—C16	102.25 (17)	H19A—C19—H19B	109.5
C20—C15—H15	108.8	C18—C19—H19C	109.5
C14—C15—H15	108.8	H19A—C19—H19C	109.5
C16—C15—H15	108.8	H19B—C19—H19C	109.5
N1—C14—C15	107.9 (2)	C10'—C11'—C1'	100.6 (9)
N1'—C14—C15	103.6 (3)	C10'—C11'—N1'	115.5 (11)
N1—C14—H14A	110.1	C1'—C11'—N1'	105.8 (10)
N1'—C14—H14A	141.2	C10'—C11'—C16	106.9 (11)
C15—C14—H14A	110.1	C1'—C11'—C16	123.8 (12)
N1—C14—H14B	110.1	N1'—C11'—C16	104.9 (8)
N1'—C14—H14B	76.0	C2'—C1'—C12'	113.3 (11)
C15—C14—H14B	110.1	C2'—C1'—C11'	136.2 (10)
H14A—C14—H14B	108.4	C12'—C1'—C11'	110.4 (12)
N1—C14—H14C	109.8	C1'—C2'—C3'	123.3 (8)
N1'—C14—H14C	141.0	C1'—C2'—H2'	118.3
C15—C14—H14C	110.1	C3'—C2'—H2'	118.3
H14B—C14—H14C	108.7	C2'—C3'—C4'	119.8 (9)
N1—C14—H14D	110.3	C2'—C3'—H3'	120.1
N1'—C14—H14D	76.3	C4'—C3'—H3'	120.1
C15—C14—H14D	110.1	C5'—C4'—C3'	121.1 (9)
H14A—C14—H14D	108.2	C5'—C4'—H4'	119.5
H14C—C14—H14D	108.5	C3'—C4'—H4'	119.5
O2—C17—O3'	121.8 (9)	C6'—C5'—C4'	127.0 (8)
O2'—C17—O3'	124.1 (11)	C6'—C5'—C12'	118.0 (9)
O2—C17—O3	121.3 (8)	C4'—C5'—C12'	115.0 (9)
O2'—C17—O3	126.2 (10)	C5'—C6'—C7'	120.2 (9)
O2—C17—C16	128.0 (6)	C5'—C6'—H6'	119.9
O2'—C17—C16	123.4 (10)	C7'—C6'—H6'	119.9
O3'—C17—C16	109.8 (7)	C6'—C7'—C8'	122.1 (8)
O3—C17—C16	109.2 (5)	C6'—C7'—H7'	119.0
O4—C20—C24	109.18 (18)	C8'—C7'—H7'	119.0
O4—C20—C15	107.62 (16)	C9'—C8'—C7'	118.1 (8)
C24—C20—C15	116.58 (19)	C9'—C8'—H8'	121.0
O4—C20—H20	107.7	C7'—C8'—H8'	121.0
C24—C20—H20	107.7	C8'—C9'—C12'	119.3 (11)
C15—C20—H20	107.7	C8'—C9'—C10'	134.6 (12)
O4—C21—O5	110.61 (19)	C12'—C9'—C10'	106.1 (10)
O4—C21—C22	114.25 (19)	C5'—C12'—C9'	122.4 (9)
O5—C21—C22	104.42 (19)	C5'—C12'—C1'	127.3 (15)
O4—C21—H21	109.1	C9'—C12'—C1'	110.3 (14)
O5—C21—H21	109.1	O1'—C10'—C9'	123.5 (12)
C22—C21—H21	109.1	O1'—C10'—C11'	124.5 (10)
O6—C22—C23	108.2 (2)	C9'—C10'—C11'	111.9 (10)
O6—C22—C21	103.96 (19)	C14—N1'—C11'	106.5 (5)
C23—C22—C21	114.76 (19)	C14—N1'—C13'	105.0 (8)
O6—C22—H22	109.9	C11'—N1'—C13'	111.4 (9)
C23—C22—H22	109.9	N1'—C13'—H13D	109.5
C21—C22—H22	109.9	N1'—C13'—H13E	109.5
O8—C23—C22	107.2 (2)	H13D—C13'—H13E	109.5
O8—C23—C24	104.16 (18)	N1'—C13'—H13F	109.5
C22—C23—C24	115.87 (18)	H13D—C13'—H13F	109.5
O8—C23—H23	109.8	H13E—C13'—H13F	109.5
C22—C23—H23	109.8	C17—O3'—C18'	114.3 (12)
C24—C23—H23	109.8	O3'—C18'—C19'	105.5 (12)
O7—C24—C20	111.12 (17)	O3'—C18'—H18C	110.6
O7—C24—C23	104.28 (19)	C19'—C18'—H18C	110.6
C20—C24—C23	112.02 (18)	O3'—C18'—H18D	110.6
O7—C24—H24	109.8	C19'—C18'—H18D	110.6
C20—C24—H24	109.8	H18C—C18'—H18D	108.8
C23—C24—H24	109.8	C18'—C19'—H19D	109.5
O7—C28—O8	104.78 (17)	C18'—C19'—H19E	109.5
O7—C28—C30	114.7 (12)	H19D—C19'—H19E	109.5
O8—C28—C30	105.8 (11)	C18'—C19'—H19F	109.5
O7—C28—C30'	99.3 (19)	H19D—C19'—H19F	109.5
O8—C28—C30'	111.0 (17)	H19E—C19'—H19F	109.5
O7—C28—C29'	119 (2)	O5—C25—O6	103.7 (3)
O8—C28—C29'	109.5 (17)	O5—C25—C26	106.3 (7)
C30—C28—C29'	102 (2)	O6—C25—C26	106.1 (6)
C30'—C28—C29'	113 (2)	O5—C25—C27	111.5 (5)
O7—C28—C29	105.6 (10)	O6—C25—C27	115.7 (7)
O8—C28—C29	110.5 (9)	C26—C25—C27	112.7 (5)
C30—C28—C29	115.1 (13)	C25—C26—H26A	109.5
C30'—C28—C29	123.2 (19)	C25—C26—H26B	109.5
C21—O4—C20	113.90 (16)	H26A—C26—H26B	109.5
C21—O5—C25	108.1 (3)	C25—C26—H26C	109.5
C21—O5—C25'	113.4 (4)	H26A—C26—H26C	109.5
C22—O6—C25	103.9 (3)	H26B—C26—H26C	109.5
C22—O6—C25'	112.7 (4)	C25—C27—H27A	109.5
C28—O7—C24	109.05 (18)	C25—C27—H27B	109.5
C23—O8—C28	106.4 (2)	H27A—C27—H27B	109.5
N1—C11—C1	120.7 (11)	C25—C27—H27C	109.5
N1—C11—C10	109.2 (8)	H27A—C27—H27C	109.5
C1—C11—C10	104.3 (8)	H27B—C27—H27C	109.5
N1—C11—C16	99.8 (7)	O5—C25'—O6	102.7 (6)
C1—C11—C16	108.2 (10)	O5—C25'—C26'	117.8 (12)
C10—C11—C16	115.2 (10)	O6—C25'—C26'	118.3 (12)
C2—C1—C12	117.8 (11)	O5—C25'—C27'	106.4 (11)
C2—C1—C11	134.5 (10)	O6—C25'—C27'	98.9 (11)
C12—C1—C11	107.7 (11)	C26'—C25'—C27'	110.6 (11)
C1—C2—C3	119.8 (8)	C25'—C26'—H26D	109.5
C1—C2—H2	120.1	C25'—C26'—H26E	109.5
C3—C2—H2	120.1	H26D—C26'—H26E	109.5
C4—C3—C2	122.6 (8)	C25'—C26'—H26F	109.5
C4—C3—H3	118.7	H26D—C26'—H26F	109.5
C2—C3—H3	118.7	H26E—C26'—H26F	109.5
C3—C4—C5	119.4 (9)	C25'—C27'—H27D	109.5
C3—C4—H4	120.3	C25'—C27'—H27E	109.5
C5—C4—H4	120.3	H27D—C27'—H27E	109.5
C6—C5—C4	128.4 (8)	C25'—C27'—H27F	109.5
C6—C5—C12	114.9 (9)	H27D—C27'—H27F	109.5
C4—C5—C12	116.7 (8)	H27E—C27'—H27F	109.5
C5—C6—C7	123.7 (9)	C28—C29—H29A	109.5
C5—C6—H6	118.1	C28—C29—H29B	109.5
C7—C6—H6	118.1	H29A—C29—H29B	109.5
C8—C7—C6	119.8 (10)	C28—C29—H29C	109.5
C8—C7—H7	120.1	H29A—C29—H29C	109.5
C6—C7—H7	120.1	H29B—C29—H29C	109.5
C7—C8—C9	119.9 (9)	C28—C30—H30A	109.5
C7—C8—H8	120.0	C28—C30—H30B	109.5
C9—C8—H8	120.0	H30A—C30—H30B	109.5
C8—C9—C12	119.3 (11)	C28—C30—H30C	109.5
C8—C9—C10	135.8 (11)	H30A—C30—H30C	109.5
C12—C9—C10	104.9 (9)	H30B—C30—H30C	109.5
C1—C12—C5	123.6 (13)	C28—C29'—H29D	109.5
C1—C12—C9	114.1 (14)	C28—C29'—H29E	109.5
C5—C12—C9	122.3 (10)	H29D—C29'—H29E	109.5
O1—C10—C9	124.9 (10)	C28—C29'—H29F	109.5
O1—C10—C11	126.0 (9)	H29D—C29'—H29F	109.5
C9—C10—C11	108.9 (8)	H29E—C29'—H29F	109.5
C14—N1—C11	112.2 (5)	C28—C30'—H30D	109.5
C14—N1—C13	109.5 (8)	C28—C30'—H30E	109.5
C11—N1—C13	107.2 (8)	H30D—C30'—H30E	109.5
N1—C13—H13A	109.5	C28—C30'—H30F	109.5
N1—C13—H13B	109.5	H30D—C30'—H30F	109.5
H13A—C13—H13B	109.5	H30E—C30'—H30F	109.5
N1—C13—H13C	109.5		
			
C17—C16—C15—C20	−82.5 (3)	C12—C9—C10—C11	−3.1 (17)
C11—C16—C15—C20	146.3 (4)	N1—C11—C10—O1	−50.4 (17)
C11'—C16—C15—C20	157.3 (5)	C1—C11—C10—O1	179.3 (14)
C17—C16—C15—C14	152.2 (2)	C16—C11—C10—O1	60.8 (18)
C11—C16—C15—C14	21.0 (5)	N1—C11—C10—C9	133.7 (11)
C11'—C16—C15—C14	32.0 (5)	C1—C11—C10—C9	3.4 (16)
C20—C15—C14—N1	−123.3 (3)	C16—C11—C10—C9	−115.1 (12)
C16—C15—C14—N1	−1.8 (4)	N1'—C14—N1—C11	69.9 (6)
C20—C15—C14—N1'	−163.6 (3)	C15—C14—N1—C11	−19.8 (7)
C16—C15—C14—N1'	−42.1 (4)	N1'—C14—N1—C13	−49.0 (8)
C15—C16—C17—O2	−16.5 (11)	C15—C14—N1—C13	−138.7 (6)
C11—C16—C17—O2	110.0 (12)	C1—C11—N1—C14	−86.4 (10)
C11'—C16—C17—O2	101.5 (12)	C10—C11—N1—C14	152.9 (7)
C15—C16—C17—O2'	8.5 (12)	C16—C11—N1—C14	31.7 (8)
C11—C16—C17—O2'	135.0 (13)	C1—C11—N1—C13	33.8 (12)
C11'—C16—C17—O2'	126.4 (13)	C10—C11—N1—C13	−86.9 (10)
C15—C16—C17—O3'	170.4 (10)	C16—C11—N1—C13	151.9 (8)
C11—C16—C17—O3'	−63.0 (11)	O2—C17—O3—C18	3.6 (16)
C11'—C16—C17—O3'	−71.6 (11)	O2'—C17—O3—C18	−20.7 (18)
C15—C16—C17—O3	177.0 (6)	O3'—C17—O3—C18	−93 (9)
C11—C16—C17—O3	−56.4 (8)	C16—C17—O3—C18	171.2 (8)
C11'—C16—C17—O3	−65.0 (9)	C17—O3—C18—C19	−174.7 (12)
C14—C15—C20—O4	53.0 (2)	C17—C16—C11'—C10'	102.4 (10)
C16—C15—C20—O4	−63.7 (2)	C15—C16—C11'—C10'	−133.6 (10)
C14—C15—C20—C24	−70.0 (3)	C11—C16—C11'—C10'	−32 (6)
C16—C15—C20—C24	173.3 (2)	C17—C16—C11'—C1'	−13.4 (15)
O4—C21—C22—O6	−104.8 (2)	C15—C16—C11'—C1'	110.6 (13)
O5—C21—C22—O6	16.2 (3)	C11—C16—C11'—C1'	−148 (7)
O4—C21—C22—C23	13.2 (3)	C17—C16—C11'—N1'	−134.5 (6)
O5—C21—C22—C23	134.1 (2)	C15—C16—C11'—N1'	−10.5 (8)
O6—C22—C23—O8	−166.06 (18)	C11—C16—C11'—N1'	91 (6)
C21—C22—C23—O8	78.4 (3)	C10'—C11'—C1'—C2'	177 (2)
O6—C22—C23—C24	78.2 (2)	N1'—C11'—C1'—C2'	57 (2)
C21—C22—C23—C24	−37.3 (3)	C16—C11'—C1'—C2'	−64 (3)
O4—C20—C24—O7	−74.5 (2)	C10'—C11'—C1'—C12'	−7.6 (19)
C15—C20—C24—O7	47.7 (3)	N1'—C11'—C1'—C12'	−128.1 (14)
O4—C20—C24—C23	41.7 (2)	C16—C11'—C1'—C12'	111.2 (16)
C15—C20—C24—C23	163.89 (19)	C12'—C1'—C2'—C3'	2 (2)
O8—C23—C24—O7	11.9 (2)	C11'—C1'—C2'—C3'	177.6 (18)
C22—C23—C24—O7	129.3 (2)	C1'—C2'—C3'—C4'	−5.6 (18)
O8—C23—C24—C20	−108.4 (2)	C2'—C3'—C4'—C5'	5.0 (15)
C22—C23—C24—C20	9.0 (3)	C3'—C4'—C5'—C6'	177.7 (12)
O5—C21—O4—C20	−76.6 (2)	C3'—C4'—C5'—C12'	−1.4 (14)
C22—C21—O4—C20	40.9 (3)	C4'—C5'—C6'—C7'	−177.2 (9)
C24—C20—O4—C21	−70.7 (2)	C12'—C5'—C6'—C7'	1.9 (18)
C15—C20—O4—C21	161.89 (17)	C5'—C6'—C7'—C8'	0.2 (17)
O4—C21—O5—C25	132.1 (5)	C6'—C7'—C8'—C9'	−2.6 (15)
C22—C21—O5—C25	8.8 (5)	C7'—C8'—C9'—C12'	3 (2)
O4—C21—O5—C25'	111.7 (10)	C7'—C8'—C9'—C10'	−177.9 (19)
C22—C21—O5—C25'	−11.6 (10)	C6'—C5'—C12'—C9'	−2 (2)
C23—C22—O6—C25	−157.0 (4)	C4'—C5'—C12'—C9'	177.5 (16)
C21—C22—O6—C25	−34.6 (5)	C6'—C5'—C12'—C1'	178.9 (17)
C23—C22—O6—C25'	−138.5 (9)	C4'—C5'—C12'—C1'	−2 (2)
C21—C22—O6—C25'	−16.1 (10)	C8'—C9'—C12'—C5'	−1 (3)
O8—C28—O7—C24	−26.9 (3)	C10'—C9'—C12'—C5'	179.8 (16)
C30—C28—O7—C24	−142.4 (11)	C8'—C9'—C12'—C1'	178.9 (16)
C30'—C28—O7—C24	−141.7 (17)	C10'—C9'—C12'—C1'	−1 (2)
C29'—C28—O7—C24	96 (2)	C2'—C1'—C12'—C5'	1 (3)
C29—C28—O7—C24	89.8 (10)	C11'—C1'—C12'—C5'	−175.0 (17)
C20—C24—O7—C28	130.0 (2)	C2'—C1'—C12'—C9'	−178.1 (17)
C23—C24—O7—C28	9.2 (2)	C11'—C1'—C12'—C9'	6 (2)
C22—C23—O8—C28	−151.80 (19)	C8'—C9'—C10'—O1'	−5 (3)
C24—C23—O8—C28	−28.5 (2)	C12'—C9'—C10'—O1'	174.4 (19)
O7—C28—O8—C23	34.8 (2)	C8'—C9'—C10'—C11'	176 (2)
C30—C28—O8—C23	156.3 (11)	C12'—C9'—C10'—C11'	−4 (2)
C30'—C28—O8—C23	141 (2)	C1'—C11'—C10'—O1'	−171.6 (18)
C29'—C28—O8—C23	−94 (2)	N1'—C11'—C10'—O1'	−58 (2)
C29—C28—O8—C23	−78.5 (12)	C16—C11'—C10'—O1'	58 (2)
C17—C16—C11—N1	−161.8 (4)	C1'—C11'—C10'—C9'	7 (2)
C15—C16—C11—N1	−31.4 (7)	N1'—C11'—C10'—C9'	120.7 (15)
C11'—C16—C11—N1	−113 (7)	C16—C11'—C10'—C9'	−123.1 (15)
C17—C16—C11—C1	−34.8 (12)	N1—C14—N1'—C11'	−65.4 (6)
C15—C16—C11—C1	95.6 (10)	C15—C14—N1'—C11'	36.3 (7)
C11'—C16—C11—C1	14 (6)	N1—C14—N1'—C13'	52.9 (8)
C17—C16—C11—C10	81.5 (9)	C15—C14—N1'—C13'	154.6 (7)
C15—C16—C11—C10	−148.1 (8)	C10'—C11'—N1'—C14	101.7 (9)
C11'—C16—C11—C10	130 (7)	C1'—C11'—N1'—C14	−148.0 (8)
N1—C11—C1—C2	51 (2)	C16—C11'—N1'—C14	−15.7 (9)
C10—C11—C1—C2	174.5 (17)	C10'—C11'—N1'—C13'	−12.3 (12)
C16—C11—C1—C2	−62 (2)	C1'—C11'—N1'—C13'	98.0 (11)
N1—C11—C1—C12	−125.4 (12)	C16—C11'—N1'—C13'	−129.7 (8)
C10—C11—C1—C12	−2.3 (17)	O2—C17—O3'—C18'	7 (2)
C16—C11—C1—C12	120.8 (13)	O2'—C17—O3'—C18'	−17 (2)
C12—C1—C2—C3	−3.6 (19)	O3—C17—O3'—C18'	95 (10)
C11—C1—C2—C3	179.9 (15)	C16—C17—O3'—C18'	−179.2 (12)
C1—C2—C3—C4	0.6 (13)	C17—O3'—C18'—C19'	−136.3 (18)
C2—C3—C4—C5	2.0 (13)	C21—O5—C25—O6	−30.3 (7)
C3—C4—C5—C6	−179.6 (8)	C25'—O5—C25—O6	78.6 (14)
C3—C4—C5—C12	−1.5 (15)	C21—O5—C25—C26	−141.9 (6)
C4—C5—C6—C7	179.5 (11)	C25'—O5—C25—C26	−33.1 (14)
C12—C5—C6—C7	1.4 (15)	C21—O5—C25—C27	94.9 (5)
C5—C6—C7—C8	0.2 (19)	C25'—O5—C25—C27	−156.3 (17)
C6—C7—C8—C9	−1.2 (19)	C22—O6—C25—O5	40.5 (7)
C7—C8—C9—C12	1 (2)	C25'—O6—C25—O5	−78.8 (14)
C7—C8—C9—C10	−178.7 (18)	C22—O6—C25—C26	152.2 (6)
C2—C1—C12—C5	4 (2)	C25'—O6—C25—C26	33.0 (15)
C11—C1—C12—C5	−178.5 (15)	C22—O6—C25—C27	−81.9 (5)
C2—C1—C12—C9	−176.9 (15)	C25'—O6—C25—C27	158.8 (17)
C11—C1—C12—C9	1 (2)	C21—O5—C25'—O6	2.3 (15)
C6—C5—C12—C1	176.8 (14)	C25—O5—C25'—O6	−76.2 (14)
C4—C5—C12—C1	−2 (2)	C21—O5—C25'—C26'	−129.7 (9)
C6—C5—C12—C9	−2 (2)	C25—O5—C25'—C26'	152 (2)
C4—C5—C12—C9	179.6 (15)	C21—O5—C25'—C27'	105.7 (10)
C8—C9—C12—C1	−177.8 (15)	C25—O5—C25'—C27'	27.2 (15)
C10—C9—C12—C1	2 (2)	C22—O6—C25'—O5	9.3 (15)
C8—C9—C12—C5	1 (3)	C25—O6—C25'—O5	75.9 (13)
C10—C9—C12—C5	−179.4 (14)	C22—O6—C25'—C26'	140.9 (11)
C8—C9—C10—O1	0 (3)	C25—O6—C25'—C26'	−153 (2)
C12—C9—C10—O1	−179.1 (15)	C22—O6—C25'—C27'	−99.9 (10)
C8—C9—C10—C11	176.3 (17)	C25—O6—C25'—C27'	−33.3 (15)

### Hydrogen-bond geometry (Å, º)

**Table:**

*D*—H···*A*	*D*—H	H···*A*	*D*···*A*	*D*—H···*A*
C23—H23···O1^i^	0.98	2.45	3.324 (7)	148

Symmetry code: (i) *x*−1/2, −*y*+1/2, −*z*+1.
